# Correction of Vertical Smile Discrepancy through Ceramic Laminate Veneers and Surgical Crown Lengthening

**DOI:** 10.1155/2019/1230610

**Published:** 2019-08-14

**Authors:** Paula Bernardon, Carlos Estevão Lagustera, Luiz Roberto Coutinho Manhães Junior, Bruno de Castro Figuêiredo, Danielle Shima Luize, Rolando Plumer Pezzini, George Borja de Freitas, José Luiz Cintra Junqueira

**Affiliations:** ^1^West State University of Parana, No. 1618 Universitária St., Cascavel, PR 85819-110, Brazil; ^2^São Leopoldo Mandic College-SP, No. 13 Dr. José Rocha Junqueira St., Campinas, SP 13045-755, Brazil; ^3^Integrated College of Patos- (FIP-) PB, Horácio Nóbrega St., S/N, Belo Horizonte, Patos, PB 58704-000, Brazil; ^4^Faculdade São Leopoldo Mandic-SP, No. 13 Dr. José Rocha Junqueira St., Campinas, SP 13045-755, Brazil

## Abstract

In cases where malocclusion is associated with intrinsic discoloration and/or discrepancies in tooth size and shape, such as peg-shaped laterals, orthodontics alone may not improve the aesthetics. In these situations, veneers may be considered as an adjunct to orthodontic treatment to improve the overall aesthetics. The aim of this study is to report a clinical case where an uneven occlusal plane was corrected, and the positioning of gingival zeniths, color, shape, and size of the dental elements involved were improved by means of gingivectomy and rehabilitation with 10 ceramic laminate veneers. It was possible to conclude that multidisciplinary treatment, when properly planned and indicated, respecting the limits and established techniques of periodontics, prosthesis, and dentistry, makes small occlusal leveling predictable and possible through these tools.

## 1. Introduction

Ceramic laminates are a highly conservative treatment when compared to total crowns due to the minimum structure removal required for this procedure, around 0.3 to 0.9 mm. Ideally, the preparation should be restricted to the enamel, although dentin exposure is often unavoidable, especially in the cervical area, as discussed by Gresnigt et al. [1].

The ceramic laminates are restorations that are bonded using adhesive cements that range from the application of phosphoric acid to silanization and the application of the adhesive and the cement itself, which guarantee high predictability, as well as aesthetics, allowing improvements in color, shape, positioning, reestablishment of the vertical dimension of occlusion, and dental exposure (D'Arcangelo et al. [2]). According to Aboushelib et al. [3], “Once properly cemented, ceramic veneers become an integral part of the tooth structure and share part of applied loading stresses during masticatory cycle.”

Patients who present significant dental misalignment should have as their first choice of treatment the orthodontic movement as the most conservative option. However, those who have defects in color or shape of the dental elements will already be subject to a more invasive restorative treatment for aesthetic correction. In cases such as this, minor dental corrections can be performed during dental preparation and the gingival zenith may become harmonic through crown lengthening, according to Marchionatti et al. [4].

In order to correct or change the positioning of the gingival zeniths, some periodontal procedures may be necessary, such as surgical clinical crown lengthening or root coverage. These maneuvers will allow a proper new dental height (Patel and Durey [5]; Ganji et al. [6]).

The aim of this study is to report a clinical case where an uneven occlusal plane was corrected, the positioning of gingival zeniths, color, shape, and size of the dental elements involved were improved by means of gingivectomy and rehabilitation with 10 ceramic laminate veneers.

## 2. Case Report

This case involves a 56-year-old male patient with an angle class I occlusion, but with an uneven occlusal plane and dental elements with alterations in color, size, shape, incisal wear, and gingival zenith. Initially, dental impressions from both dental arches (performed with the addition of silicone rubber; Virtual, Ivoclar Vivadent), occlusion record, and digital smile design protocol photos (DSD) were used for the case planning (Figures 1–3). By means of radiographs, it was possible to evaluate the necessity of strategic bone removals to redefine the supracrestal space (Figure 4).

80% of the width to height ratio was applied to define the new design of the dental elements, starting at the central incisor. The possibility of performing this new aesthetic planning was confirmed by radiographic and clinical examinations and subsequent mock-ups (Figures 5(a)–5(c) and 6).

The mock-up with Protemp™ 4 bisacrylic resin was performed in the patient for aesthetic, phonetic, and functional proof of virtual planning by means of CAD/CAM milling diagnostic waxing. A surgical guide was manufactured to increase the clinical crown (gingivectomy and osteotomy) according to the parameters suggested by the virtual design (DSD) (Figure 7). According to Lee's classification system for aesthetic crown lengthening procedures (Lee [7]), all dental elements were classified as type I (sufficient soft tissue allows gingival exposure of the alveolar crest or violation of the biologic width), except for element 23 which was classified as type II (sufficient soft tissue allows gingival excision without the exposure of the alveolar crest but with violation of the biologic width).

In order to perform surgical crown lengthening (Figures 8–11), the surgical guide was used to perform the incisions and the definition of the new gingival zeniths. Osteotomies were performed intrasulcularly with the aid of Ochsenbein's chisels, acquiring a biological space of 3 mm. The patient was followed up postoperatively; because the gingival margin was stable and to avoid the inflammation process, no type of temporary material was used during the healing period. 60 days after the surgical procedure, new dental impressions were obtained, diagnostic waxing was performed under the new periodontal parameters, and a new mock-up with Protemp™ 4 Temporization Material (3M ESPE) bisacrylic resin was realized (Figure 12).

Dental preparations under gingival spacing with retractor cord number 000 Ultrapack (Ultradent) were obtained by means of the selective wear of the tooth structure with a diamond drill in an electric motor under constant irrigation, guided by a condensation silicone rubber index made from the diagnostic waxing, both palatal and incisal and vestibular (in its three-thirds). Measurements of adequate ceramic space (between 0.3 and 0.5 mm) were checked from time to time with the aid of a periodontal probe from the dental enamel to the guide (Figures 13 and 14). Every angle should be rounded, and the preparation should be adequately finished and polished. Care should be taken to keep the tooth reduction inside the thickness of the enamel (Figure 15).

After polishing and finishing the preparation, a number 2 Ultrapack retractor cord (Ultradent) was inserted into all 10 dental elements soaked in hemostatic solution and held for 5 minutes for a horizontal spacing of the gingival tissues (Figure 16).

After the cord removal, the preparations were washed, dried, and scanned using the 3Shape (TRIOS) scanner. The antagonist teeth and the occlusion of the patient were also obtained by means of the scanning. As the preparation was restricted to the dental enamel, dentin was not previously sealed and the provisional restoration was made with the Protemp™ 4 Temporization Material (3M ESPE) bisacrylic resin. The silicone index, obtained from the wax-up, was used to mold a self-curing composite material in the same morphology obtained with the previsualization mock-up. The direct provisional restoration, after intraoral finishing and polishing, was macromechanically retained on the prepared teeth until the luting session.

The ceramic laminate veneers (IPS e.max, Ivoclar Vivadent) were made to CAD/CAM impressions and painted by the technician. At first, the dry test was performed in the mouth, to check the individual adaptation of each one (Figures 17 and 18). This was followed by the wet test with the try-in pastes of the Variolink Esthetic LC System Kit e.max (Ivoclar Vivadent) and cemented under absolute isolation of the operative field and previous prophylaxis of dental elements, starting with the treatment of the lithium disilicate (IPS e.max, Ivoclar Vivadent) pieces with 10% hydrofluoric acid (Dentsply) (Figures 19 and 20) for 20 seconds, washing and drying, followed by the application of phosphoric acid Total Etch 37% (Ivoclar Vivadent) for 30 seconds (Figure 21), Monobond N (Ivoclar Vivadent) silanization (Figure 22) of two layers with heat activation for 1 minute, and application of Tetric N-Bond Universal adhesive (Ivoclar Vivadent) and Variolink Esthetic LC cement (Ivoclar Vivadent) in light color.

Dental elements were conditioned with 37% Total Etch phosphoric acid (Ivoclar Vivadent) for 30 seconds in enamel (Figure 23) and Tetric N-Bond Universal adhesive (Ivoclar Vivadent) (Figure 24) was applied followed by air jets for solvent evaporation, laying the laminates in pairs beginning with the central incisors and then ending one side at a time (Figure 25), removal of excesses with the aid of dental floss and brushes (Figure 26), photopolymerization for 40 seconds of each face (Figure 27) and followed by the application of Liquid Strip (Ivoclar Vivadent) glycerin on the edges of the restoration and new photopolymerization to inhibit the effect of oxygen in the last layer of cement.

A delicate margin of finishing and polishing was performed (Figure 28). A slight excess was removed with a box carver or a No. 12 scalpel blade; then, if necessary, low-grit diamond burs and flexible blades (40 and 15 *μ*m) were used on a reciprocating handpiece. Finally, composite polishers, cups, and synthetic brushes with diamond paste were used. Once the rubber dam was removed, occlusal relation was checked in maximum intercuspation, then in laterality and during protrusive movements (Figures 29 and 30).

A two-year follow-up was performed, and the pieces did not present fractures, discoloration, or decreased cementation. The margin preparation was sealed and the occlusion was stable. Other dental elements did not present structural wear and tear (Figures 31–34).

## 3. Discussion

Associated with a high level of dental aesthetics, the occlusal parameters must be stable and must ensure that the temporomandibular joint remains healthy, the teeth are firm, and the support structures are in a suitable condition. Otherwise, the prognosis of restorative treatment becomes unpredictable (Brea et al. [8]). In this case, all the mandibular excursion movements were performed; protrusive, lateral-lateral, and all contacts were checked; and possible premature contacts were removed, in order to guarantee occlusal stability.

According to Lee in 2004 [7], conventional protocols require a waiting period of 4 to 6 weeks for sufficient healing of the attachment apparatus prior to initiating restorative endeavors. In this case, a longer period of healing was expected due to patient availability.

“Minimal tooth reduction, esthetics, and maintenance of healthy tissues are the major advantages of conservative preparation of ceramic laminate veneers. Since ceramic is a translucent material, tooth-colored resin cement under these restorations is mainly reflected from beneath the restoration for optimal esthetics” (Çömlekoğlu et al. [9]). For these reasons, in this study a cement was selected that presented the try-in in its system to allow adequate selection of the final color of the set formed by the dental element, cement, and ceramic laminate.

In a randomized split-mouth clinical trial carried out by Gresnigt et al. [10], a comparison of the indirect resin composite and ceramic laminate veneers was performed. The results presented cover observations up to 120 months of clinical function and showed that ceramic veneers performed significantly better than the indirect composite ones.

Increased fractures and chippings were noticed up to 8 times in studies with composite laminate veneers. Debondings of composite laminate veneers occurred in the same patient whilst all laminate veneers functioned until the end of the study. Indirect resin composite material showed surface degradation and diminished gloss retention. On the other hand, all ceramic restorations remained smooth and their gloss was retained until the final follow-up. In this way, surface quality changes were more frequently observed in the composite veneer material that may require more maintenance over time (Gresnigt et al. [10]). However, despite the results obtained in this study for composites, they should not be discarded as a restorative option, since they have low cost, ease of access, and easy resolution of any intercurrences that may arise.

In agreement with this case, the inadequate periodontal contour of the restorations causes food impaction, making it difficult to control the plaque. For this reason, the alignment of the dental elements, either by means of orthodontic movement or by the association of periodontal restorative and surgical techniques that allow a balance between the white and pink aesthetics, guarantee health in all aspects in cases of mild to moderate discrepancies. In cases of crowding or severe malposition, in which the disharmony of the papillae is present, the treatment indicated is the orthodontic treatment prior to any restorative treatment (Brea et al. [8]).

## 4. Conclusion

It was possible to conclude that multidisciplinary treatment, when properly planned and indicated, respecting the limits and established techniques of periodontics, prosthesis, and dentistry, makes small occlusal leveling predictable and possible through these tools.

## Figures and Tables

**Figure 1:**
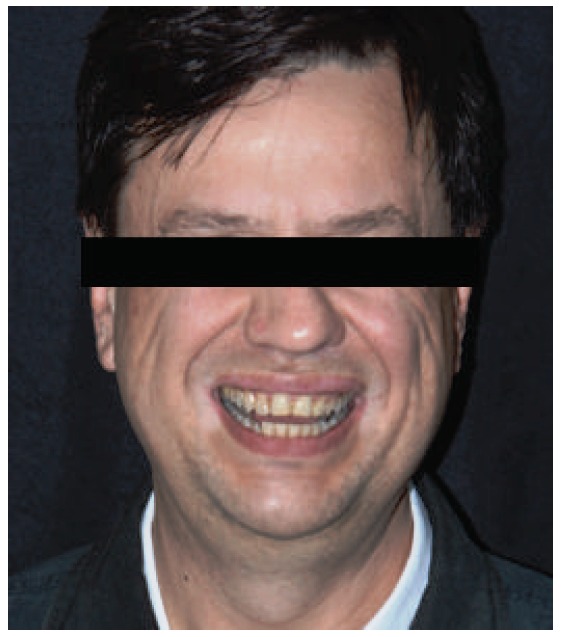
Face photograph.

**Figure 2:**
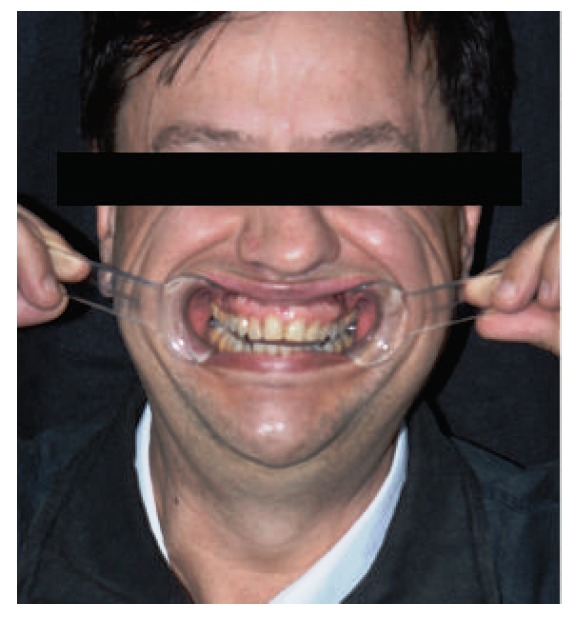
Smile with lip retractors.

**Figure 3:**
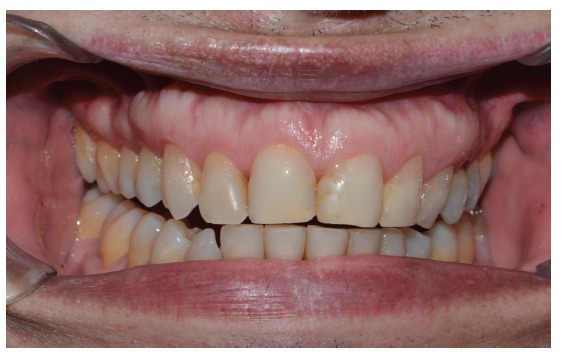
Approximate view of the smile.

**Figure 4:**
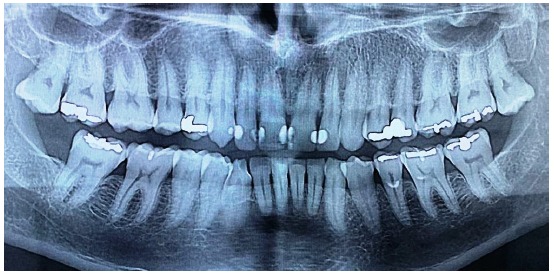
Radiograph aspect.

**Figure 5:**
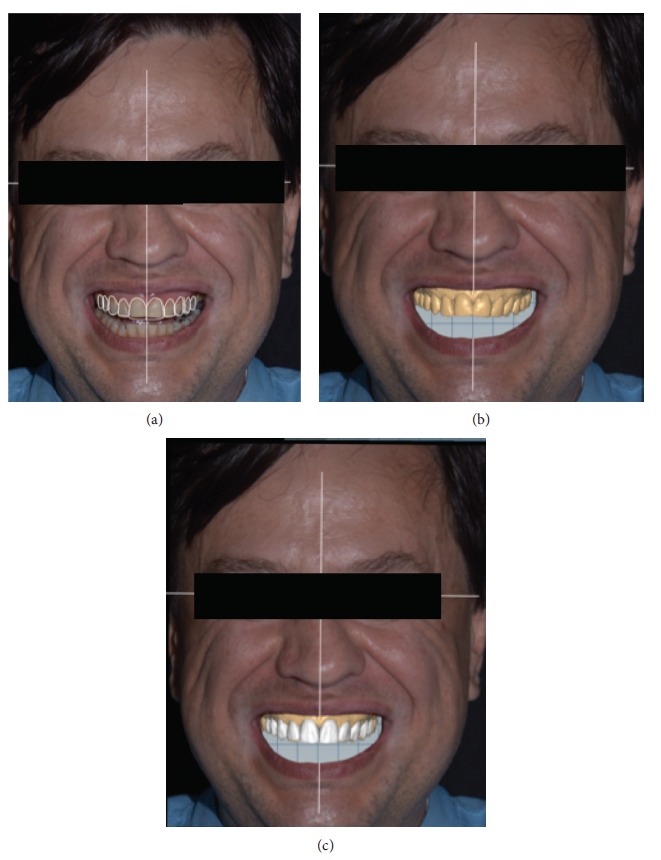
DSD planning.

**Figure 6:**
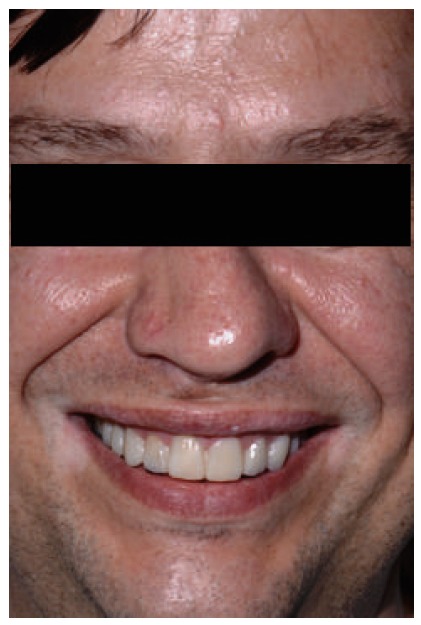
Mock-up.

**Figure 7:**
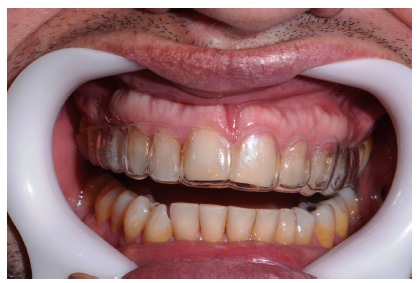
Surgical index.

**Figure 8:**
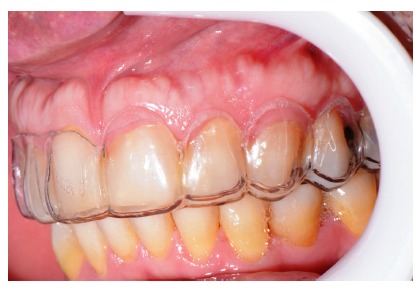
Surgical index: lateral view.

**Figure 9:**
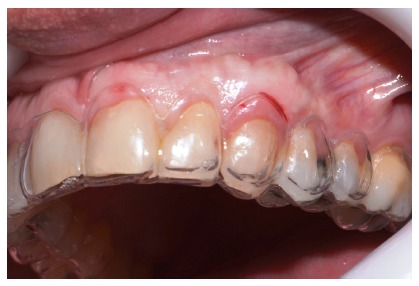
Incisions with the guide.

**Figure 10:**
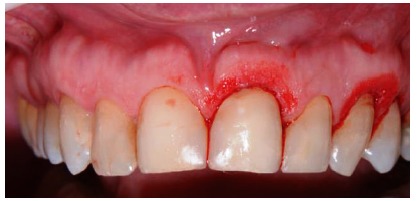
Incisions.

**Figure 11:**
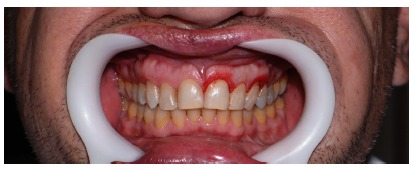
Final aspect.

**Figure 12:**
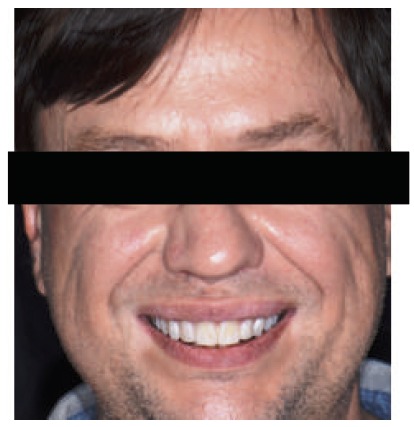
Mock up with the new gingival position.

**Figure 13:**
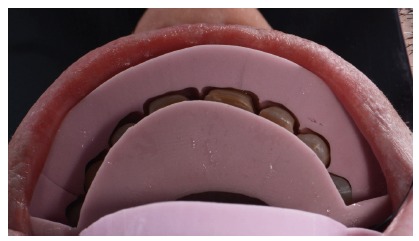
Vestibular guide.

**Figure 14:**
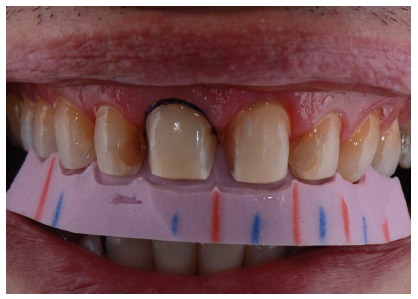
Incisal guide.

**Figure 15:**
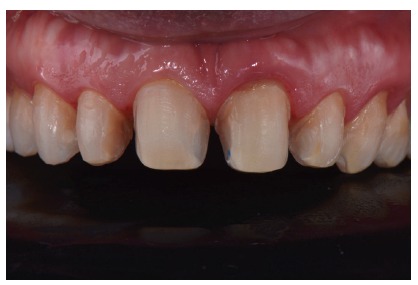
Final aspect of dental preparation.

**Figure 16:**
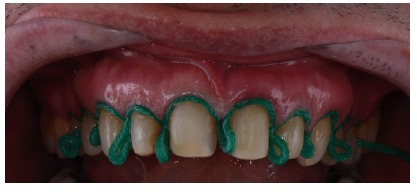
Retractor cord number 2.

**Figure 17:**
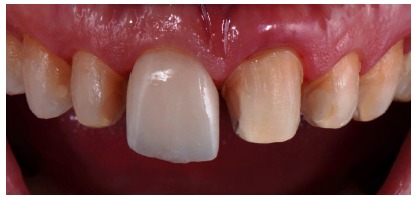
Dry proof in a vestibular view.

**Figure 18:**
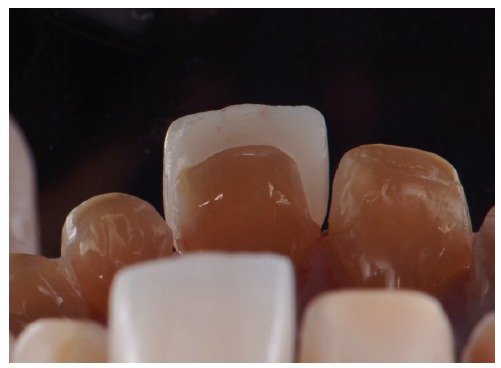
Dry proof in a palatine view.

**Figure 19:**
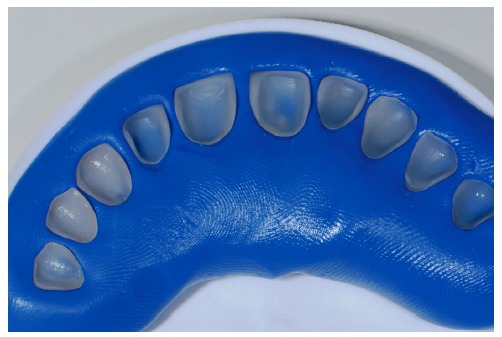
Ceramic laminate veneers.

**Figure 20:**
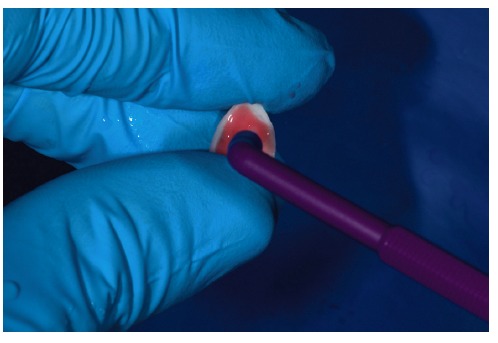
Hydrofluoric acid application for 20 seconds.

**Figure 21:**
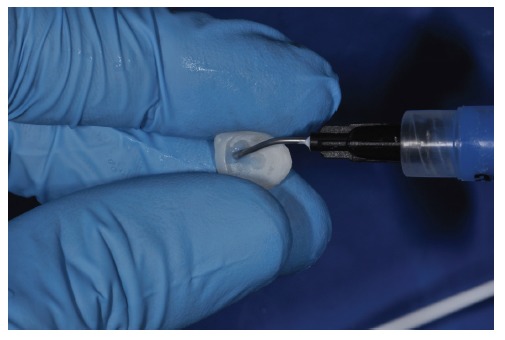
Phosphoric acid application for 30 seconds.

**Figure 22:**
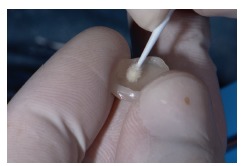
Silanization.

**Figure 23:**
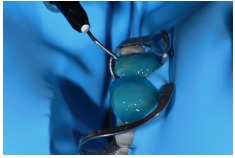
Phosphoric acid application for 30 seconds in enamel.

**Figure 24:**
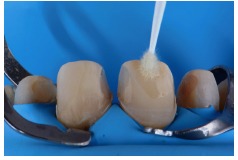
Bond application.

**Figure 25:**
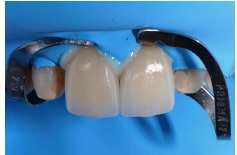
Ceramic laminate veneers with cement.

**Figure 26:**
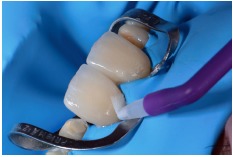
Cement excess removal.

**Figure 27:**
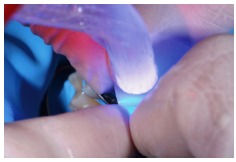
Photopolymerization.

**Figure 28:**
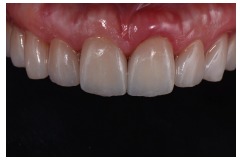
After finishing and polishing.

**Figure 29:**
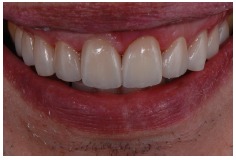
Final aspect of the smile.

**Figure 30:**
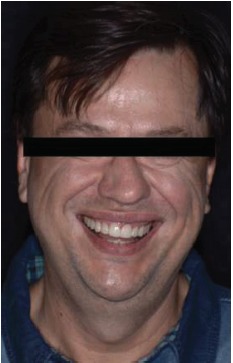
Final aspect of the face.

**Figure 31:**
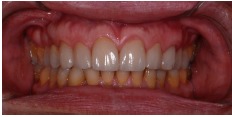
Two-year follow-up period.

**Figure 32:**
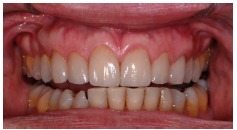
Two-year follow-up period: protrusion movement.

**Figure 33:**
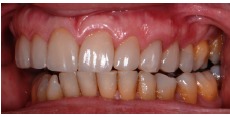
Two-year follow-up period: excursion movement.

**Figure 34:**
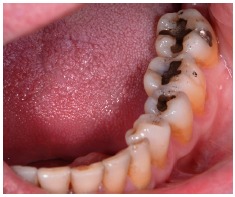
Two-year follow-up period: occlusal points in maximum intercuspidation.
